# Variant of the terrible triad of the elbow, a CASE report with a review of the literature

**DOI:** 10.1016/j.ijscr.2023.109163

**Published:** 2023-12-15

**Authors:** F. Lamnaouar, A. Rajaallah, A. Rafaoui, A. Messoudi, M. Rahmi, M. Rafai

**Affiliations:** Traumatology and Orthopedic Department P32, CHU IBN Rochd University Hospital Center, Casablanca, Morocco

**Keywords:** Elbow, Terrible triad variants, Column theory, Ring theory, Complex dislocation fractures, Instability, Stiffness

## Abstract

**Introduction:**

The terrible triad described by Hotchkiss in 1996 is a complex lesion of the elbow, following a trauma combining forced valgus and external rotation. It is a lesion that puts the elbow at risk of developing complications such as instability, stiffness, or synostosis of the proximal radio-cubital joint.

**Case report:**

We report the case of a patient who suffered a closed trauma to the right elbow following a fall onto the palm of the hand with a valgus lateral rotation mechanism. The lesion assessment showed a B2 fracture of the distal humerus (AO classification) with a line splitting the capitulum in the frontal plane, a type 3 coronoid process fracture (Morrey/Odriscoll classification), and a posterolateral elbow dislocation. The surgical treatment followed the same principles as for the terrible triad, with a reconstruction of the lateral column by osteosynthesis of the humeral palate, followed by an internal approach for osteosynthesis of the coronoid process, with the restoration of a stable elbow without laxity in the frontal plane.

**Discussion:**

On the basis of the lesion mechanism, column theory, and the schematization of the constituent elements of elbow stability in a ring, certain lesions can be placed in the same box as the terrible triad of the elbow, which also complies with the same therapeutic implications.

**Conclusion:**

Our observation underlines the possibility of the existence of lesions other than those described by Hotchkiss, which would have the same consequences: an unstable elbow with the risk of evolving into chronic instability or stiffness and whose management accepts the same management.

## Highlights

• The elbow is very congruent joint with a risk of devlopping a stiffness. The conceptualization of stability of the elbow into 4 columns forming a ring allow to put some lesions in the same box as terrible triad
• The treatment of the terrible triad goes in the sense of the lesion occurring according to Horii circle
• The conceptualization of stability of the elbow into 4 columns forming a ring allow to put some lesions in the same box as terrible triad

## 1 Introduction

Complex fracture dislocation of the elbow is a challenging condition and constitutes a diagnostic, therapeutic and prognostic problem. The elbow is considered to be an unforgiving joint, given two major risks: stiffness and instability [1]. The risk of stiffness is present due to its highly congruent bony anatomy, its relatively confined joint space, its closely stabilizing collateral ligament complex and the close relationship between the surrounding muscles, which act as secondary stabilizers [2].

In the terrible triad, the energy dissipates along a very precise path, described as Horii's circle, resulting in a fracture of the radial head, a fracture of the coronoid process and a posterior dislocation of the elbow. However, depending on the extent of the traumatic energy and the direction in which it dissipates, lesions may affect other columns, giving rise to additional or superadded lesions. The most frequent mechanism is a fall onto the palm of the hand with a combination of axial and valgus compression on the elbow and supination of the forearm in relation to the humerus [3].

A clear understanding of the anatomy and biomechanics of the elbow joint, in particular the multiple bony and ligamentous structures that contribute to joint stability, is necessary for surgical management. Restoring stability to allow early mobilization and avoiding stiffness are the main aims of treatment [1].

## 2 Case report

The reporting of this work follows the SCARE checklist criteria [16], ensuring adherence to guidelines for quality reporting in case series.

**Fig. 1:**
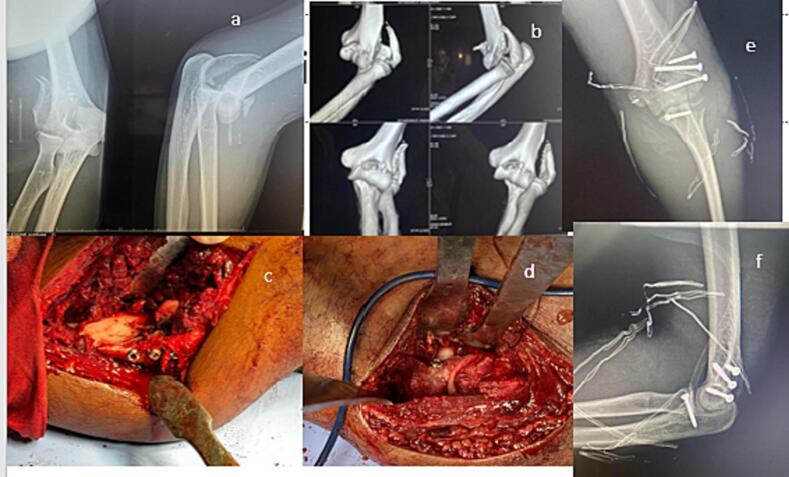
a. Radiological assessment on admission b. 3D reconstruction of the dislocated elbow fracture c. External approach with osteosynthesis of the lateral epicondyle d. Internal approach with osteosynthesis of the coronoid e, f Control X-ray.

We report the case of a 28-year-old patient who fell from a motorbike and landed on the palm of his right hand, resulting in trauma to the elbow. On admission, he had total functional impotence, a swollen elbow, and altered bony landmarks of the elbow, with no opening of the skin and no downstream vascular or -nervous disorders. Radiological findings: a dislocated fracture of the elbow combined with a fracture of the distal humerus classified as B2, a fracture of the coronoid process type 3 according to Morrey's classification with posterolateral dislocation of the elbow (Fig. 1a). A reduction maneuver was attempted but failed. On CT scan, the lateral column line detached the lateral epicondyle and continued on the capitulum, creating a frontal line and a type 3 fracture of the coronoid process according to Odriscoll's classification (Fig. 1b).

The patient was treated surgically on emergency using a lateral Kaplan, which enabled the epicondyle fracture to be reduced proximally, and fixed with three screws (we didn't have a plate in our disposition) with the re-establishment of the contact condyle- radial head. A medial approach allowed osteosynthesis of the coronoid process and reinsertion of the medial capsule-ligament plane using an anchor which allows the repair of the collateral medial ligament. After osteosynthesis and reinsertion, the elbow was stable and free of valgus or varus laxity, tested at 30 % flexion (Fig. 1c–d).

**Fig. 2:**
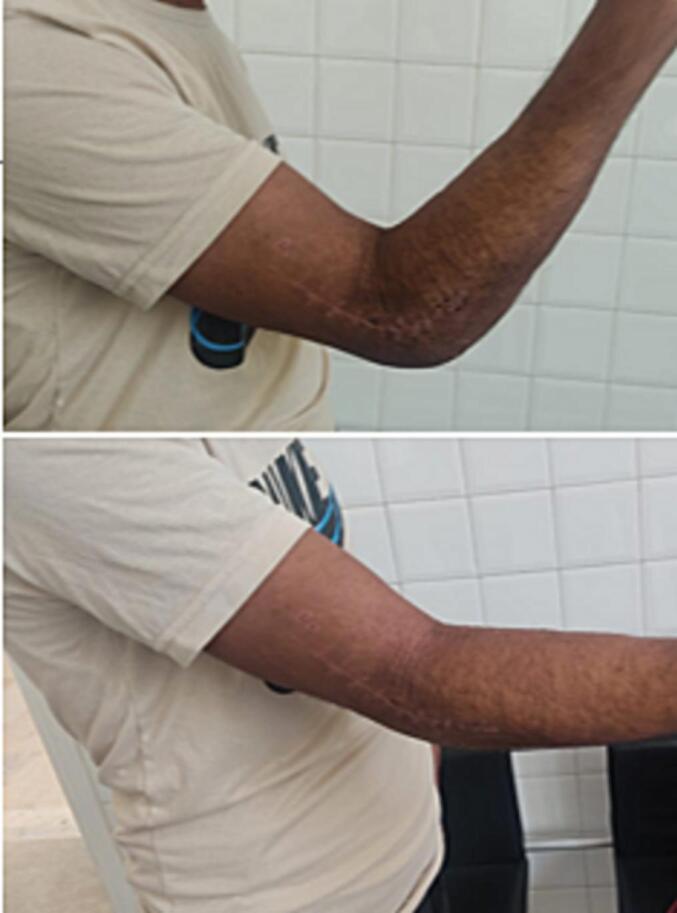
Clinical checkup 6 weeks after trauma.

**Fig. 3:**
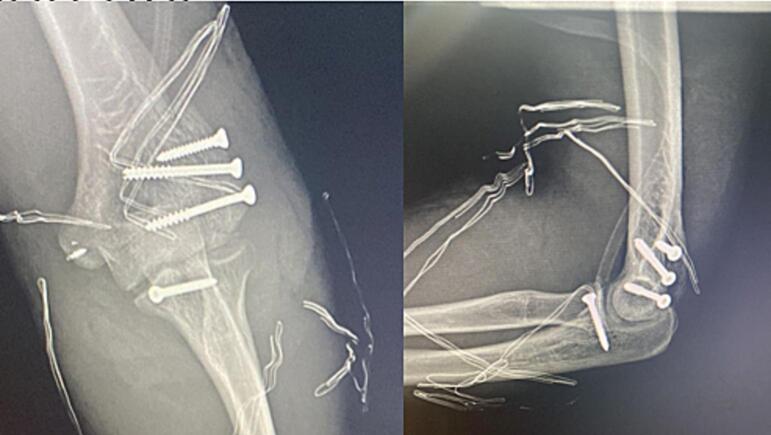
The post operative X ray images of the patient.

The immobilization was realized by an articulated orthosis, and the recovery of the range of motion was started after the 4th week (Fig. 2) with a satisfying radiologic control (Fig. 3).

**Fig. 4:**
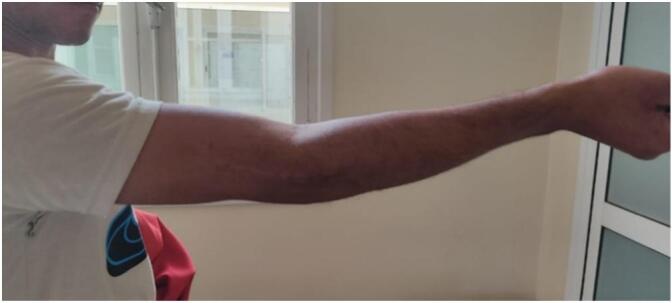
Extension at −10°/flexion 140° measured 4 months after surgery.

The follow-up of the patient at 4 months revealed good evolution with incomplete recuperation of the room of motion of the flexion-extension [−10°−0°−120°] (Fig. 4) the mayo elbow performance score was judged excellent >90, the patient did go back to his work as a mason.

## 3 Discussion

The function of the elbow is to facilitate the positioning of the hand in space through flexion extension. To enable this, the elbow has a ginglymoid huméro-ulnar joint. The key structure is the coronoid process, which is made up of 3 structures: the coronoid tubercle (sublime) and two anterolateral and anteromedial facets. In addition to being a capsulo-ligamentary insertion site, it forms a buttress that prevents posterior dislocation of the elbow [1].

The humeroulnar joint is very congruent; however, according to Kapandji [4], there is a predisposition to dislocation in extension either by forces that exert a distraction because the arc formed by the ulnar incisure forms an arc inferior to 180° and that holding will fail in the absence of soft parts. Resistance will be provided by ligamentous and muscular elements. On the other hand, there is also a predisposition to dislocation by compressive forces, and resistance is provided by bony elements such as the coronoid process and the radial head. These compressive phenomena are the most often encountered in elbow dislocations [4].

The radial head is responsible for transmitting 60 % of axial stresses and 30 % of resistance to valgus loads, while the medial collateral ligament absorbs the majority of stresses due to physiological valgus ulna and the action of periarticular muscles, which increase pressure on articular surfaces [5]. Isometric flexion against resistance can generate stresses equal to up to 4 times body weight [4].

**Fig. 5:**
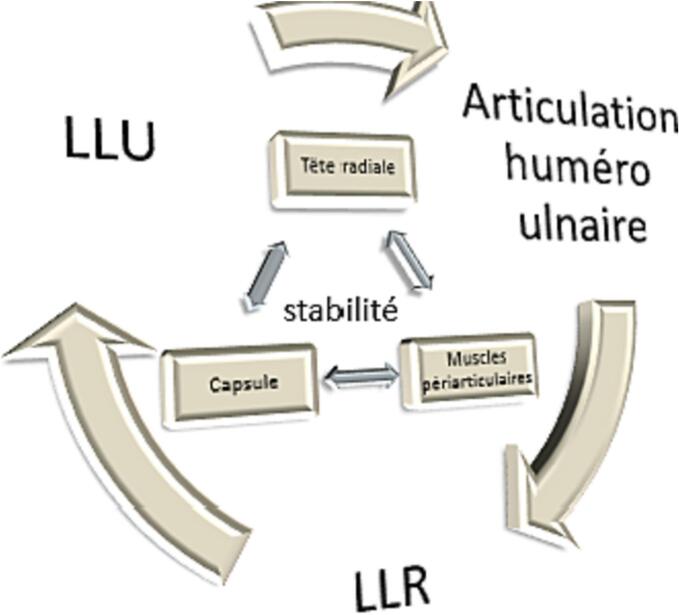
Fortress diagram of the stability elements of the elbow according to Odriscoll [6].

**Fig. 6:**
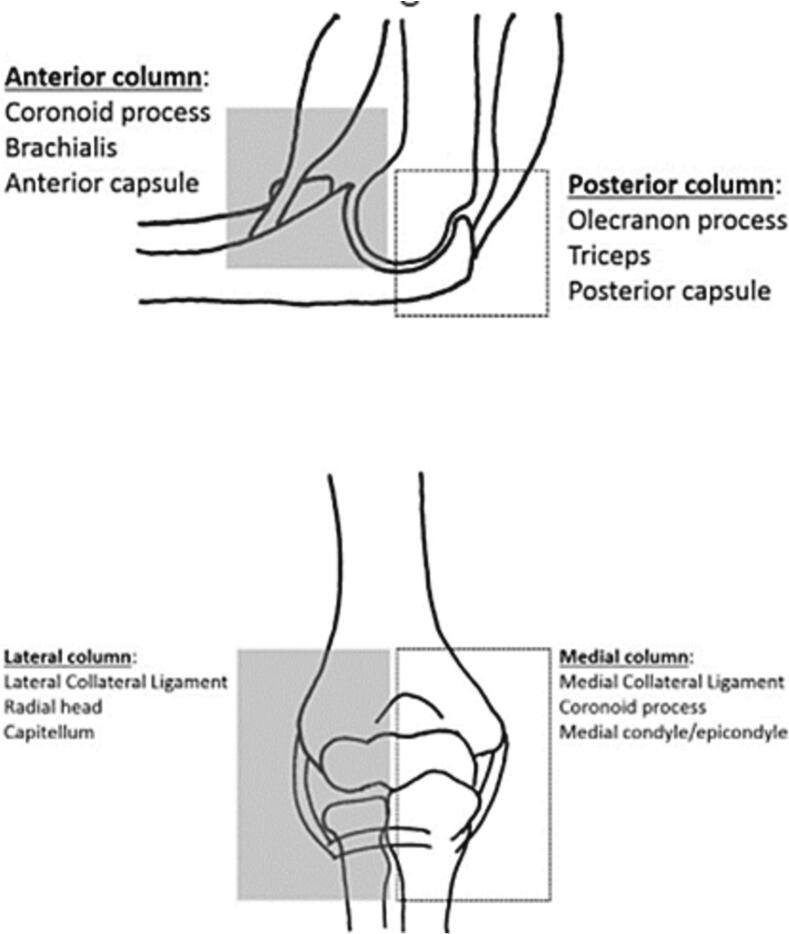
The constituents of the four columns of the elbow [3].
  In this circle, the stability of the elbow is considered to be ensured by four columns. In elbow trauma, the greater the number of elements and possibly columns affected, the more the stability of the elbow will be compromised [8]. Each of these columns is necessary to preserve stability and the arc of mobility [9]. Instability leads to chronic decentralization of the joint. Over time, this results in reduced mobility due to capsular and muscular contractures and arthrofibrosis [2].

Several biomechanical theories explain the elements responsible for elbow stability:

- The fortress theory (Fig. 5) according to Odriscoll, where the stabilizing elements are divided into primary stabilizers, which are the huméro-ulnar joint, the medial collateral ligament, and the lateral external ligament, and secondary stabilizers, which are the radial head, the capsule and the periarticular muscles, which exert a compressive effect on the joint. In the event of a coronoid lesion, the radial head becomes a crucial element in stability, and it can only be resected if the coronoid and the LLI are repaired [6].

- In the second representation (Fig. 6), similar to the ankle and pelvic ring, the elements ensuring the stability of the elbow form a ring with two imperatives: the first is that any rupture of the circle at a given point must most likely be accompanied by another on the opposite side. The second is defined as an alternative mode of failure, where injury to one of the elements of the ring must spare the element next to it; for example, fracture of the coronoid process will protect the ulnar collateral ligament [7]. In the case of our patient, the alternative mode of failure explains that the fracture of the lateral epicondyle and the condyle protected the radial head.

In 1992, O'driscoll carried out a detailed study of the dislocation process, concluding that supination stress coupled with valgus stress led to the rupture of the lateral collateral ligament and the posterolateral part of the capsule, ending in dislocation. Based on these findings, Horii and Odriscoll developed a theory of the “Horii circle”, similar to Mayfield's diagram for the carpus, in which soft tissue damage occurs from lateral to medial [10]. Three stages are then described (defined by the spectrum of instability for O'driscoll) [11]:

- Stage 1: Partial or complete rupture of the lateral collateral ligament = posterolateral subluxation
- Stage 2: Rupture of the anterior and posterior soft tissues = complete dislocation of the elbow (pronated forearm stabilizes the elbow by action of the LLU)
- Stage 3 is subdivided into three categories:
▪ 3A: Associated with a fracture of the radial head and coronoid/anterior fascicle of the LLI intact: no subluxation during the varus/valgus test
▪ 3B: The medial ligament complex is ruptured/the elbow is unstable even after reduction. A certain degree of flexion is necessary to maintain the reduced elbow (30 to 45°).
▪ 3C: humerus stripped of all soft tissue/elbow unstable even with 90° plaster cast immobilization. Flexion >90° is necessary to maintain the reduced elbow [11].

For O'driscoll, the aim of treatment is to restore bone stresses and thus convert the lesion into a simple dislocation [6].

The management of a terrible triad is currently codified [12,13,14] and is based on the following principles:

- The risk of reluxation is reduced by fixation or replacement of the radial head, reinsertion of the external collateral ligament on the epicondyle, and, if necessary, repair of the coronoid process.
- Restoring capitulo-radial contact is a key element in restoring elbow stability
- As long as the elbow remains reduced, the ulnar collateral ligament will be able to heal.
- If repair of the coronoid, radial head, and external collateral ligament does not prevent dislocation, repair of the medial collateral ligament will be considered.

A review of the literature [3] looked at variants of the terrible triad. The criteria for inclusion in the terrible triad category and its variants were the mechanism of injury and the existence of associated ligamentous injuries of the elbow and retained the article by Desai et al. [8] and Kumar et al. [15], where the injury involved the capitulum and not the radial head. Our team considered our observation to be an equivalent lesion or a variant of the terrible triad, given:

- The mechanism of injury combines valgus axial compression and supination
- An injury to the inside of the external column that, according to the ring theory, the injury to one element should protect the element next to it.
- In the principles of treatment, the stabilizing element is the capitellar-radial contact: the capitulum, like the radial head, also constitutes a stabilizing element, the excision of which, associated with an internal ligament injury, gives rise to laxity and instability of the elbow.

## 4 Conclusion

The elbow is a joint that always poses problems from a therapeutic point of view, particularly given its tendency to stiffen and the risk of instability, especially in the case of complex dislocation fractures of the elbow.

Despite the challenges posed by the terrible triad, a good application of anatomical and biomechanical knowledge with the application of basic surgical techniques can bring good results for patients [1].

The wide variety of lesions at this level should prompt us to consider broadening the definition of the terrible triad, especially as management is based on the same principles.

## Consent

Written informed consent was obtained from the patient for publication of this case report and accompanying images.

## Ethical approval

The case report is exempt from ethical approval at our institution, and only consent is necessary.

## Funding

This research did not receive any specific grant from funding agencies in the public, commercial, or not-for-profit sectors.

## CRediT authorship contribution statement

All the authors contributed to the study concept, data analysis and writing of the paper.

## Guarantor

Dr. Foad Lamnaouar.

## Declaration of competing interest

The authors declare no conflict of interest.
